# Knowledge and health beliefs of reproductive-age women in Alexandria about tetanus toxoid immunization

**DOI:** 10.1186/s42506-020-00049-8

**Published:** 2020-08-27

**Authors:** Azza Mehanna, Mervat H. Ali, Ibrahim Kharboush

**Affiliations:** 1 Health Administration and Behavioral Sciences Department, HIPH, Alexandria University, 165 El Horreya Avenue, Alexandria, 21561 Egypt; 2 Gynaecology and Obstetrics Department, Student University Hospital, Alexandria, Egypt; 3 Family Health Department, HIPH, Alexandria University, Alexandria, Egypt

**Keywords:** Maternal neonatal tetanus, Tetanus toxoid vaccine, HBM, Alexandria

## Abstract

**Background:**

Maternal neonatal tetanus is a substantial public health problem in many developing countries. In 2017, nearly, 30,848 newborns died of neonatal tetanus; thus, high immunization coverage remains a necessity. This study aims to assess knowledge and health beliefs of reproductive-age women in Alexandria about tetanus toxoid immunization.

**Methods:**

A cross-section survey of 700 females attending health offices in Alexandria was done using an interview questionnaire to collect data regarding women’s knowledge and beliefs about tetanus toxoid vaccine (TTV) and maternal and neonatal tetanus (MNT). Nine health offices were selected using multi-stage random sampling.

**Results:**

Most of studied women (83.6%) had poor knowledge of MNT and TTV. The highest percentage of women had low perception of susceptibility to MNT (48.0%), moderate perception of severity of MNT (57.4%) and barriers to TTV (58.9%), high perception of benefits of TTV (86.6%), and high self-efficacy in taking the vaccine (76.2%). Less than one-third of the sampled women (27.7%) were immune by (TT2+). Logistic regression models showed that the place of antenatal care, level of knowledge, perceived barriers, and socio-economic level were significant predictors of immunity status (*p* = 0.008, *p* = 0.032, *p* = 0.011, and *p* = 0.001, respectively).

**Conclusion:**

Women lacked information about MNT/TTV and may even have been discouraged by their private obstetricians from taking the vaccine. Perceived barriers to receiving tetanus toxoid vaccination were shown to be an important predictor of immunization behavior

## Introduction

Maternal neonatal tetanus remains a significant cause of neonatal and infant mortality in a number of developing countries [1, 2].

Tetanus is an acute infectious non-communicable disease caused by bacteria Clostridium tetani. Women exposed to the organism during pregnancy or within 6 weeks after delivery are liable to develop maternal tetanus. Infants born to non-immunized mothers are prone to neonatal tetanus (NT). Infection usually occurs through the unhealed umbilical cord. The majority of cases of neonatal tetanus develop symptoms during the first 3–14 days. The disease starts by loss of ability to suck, followed by generalized rigidity, and painful muscle spasms [2, 3].

The mortality of tetanus in general tends to be high in the absence of medical treatment where case fatality approaches 100%; this percentage decreases to 10–60% in the presence of hospital care, depending on the availability of intensive care facilities [2–4]. In 2017 (the latest year for which estimates are available), nearly 30,848 newborns died of neonatal tetanus [1, 5].

In June 2014, Egypt was validated among 35 countries that achieved MNT elimination [6]. The most recent record of Egyptian demographic health survey (EDHS) 2014 suggests an increase in tetanus toxoid coverage, where approximately 41% got two or more doses compared to 29% in 2013 as reported by EDHS 2013. However, it has been observed that the percentage of coverage in rural governorates as Menofiya has clearly exceeded that percentage in urban governorates as in Alexandria [7].

Needless to say, enhancing these coverage rates necessitates the majority of pregnant women are immunized against tetanus by at least two doses (TT2+) [2]. Moreover, reasons beyond reduction of immunization rates in some areas should be investigated.

The Health Belief Model (HBM) has been widely used in health behavior research [8]. It postulates that health behavior is influenced by an individual’s perceived threat of a health condition, the perceived benefits of engaging in a healthy behavior to decrease the health threat, and perceived barriers to performing healthy behavior [9]. It also assumes that a cue or trigger is needed to instigate action [10]. Self-efficacy, later added to HBM [8, 9], refers to confidence in one’s ability to successfully perform a particular behavior [9]. The HBM has been used to predict a wide variety of health-related behaviors including immunization behaviors [11–13].

In Egypt, among several studies conducted on tetanus toxoid vaccination [14–16], few have investigated women’s beliefs using the HBM. This study aims to explore knowledge and health beliefs of Egyptian women in Alexandria regarding MNT/TTV using the theoretical framework of the Health Belief Model.

## Methods

### Study design and sampling

A cross-sectional design was used to study women attending health offices (HOs) in Alexandria to vaccinate their children. The study included mothers in the reproductive-age group who delivered a baby within the previous 6 months.

The study was conducted on 700 females of reproductive age based on the assumption that knowledge regarding TTV = 32% [17], precision = 5% using alpha = 0.05, and design effect = 2. HOs were randomly selected using multi-stage random sampling technique, where four districts were chosen at random from the eight health districts in Alexandria. Nine HOs were randomly selected from the chosen districts using the proportional allocation technique depending on the population size in each district. All women attending the HOs who fulfilled the inclusion criteria and agreed to participate in the study were included.

### Data collection

A structured interview questionnaire was developed by the researcher to collect information on socio-demographic characteristics of the study participants, reproductive history, awareness and knowledge about MNT and TTV, and their beliefs about TTV and MNT based on HBM. The questionnaire was pilot tested on a sample of 70 women to evaluate its feasibility and reliability and a few revisions of the items of the HBM were made. Content validity of the tool was indicated by a panel of experts in the field of behavioral sciences and reproductive health. The internal consistency of HBM subscales and knowledge scale was determined by Cronbach’s alpha coefficient. Cronbach’s alpha coefficients were 0.87 (perceived susceptibility), 0.83 (perceived benefits), 0.79 (perceived barriers and self-efficacy), 0.72 (perceived severity), and 0.65 (knowledge). Socio-economic data were used to calculate the socio-economic score based on Fahmy et al. [18] with some modifications.

According to the WHO, women who received two or more doses of TTV (TT2+) were considered immune, while those who did not receive the vaccine or received less than two doses were considered non-immune [2, 19].

#### Awareness and knowledge of MNT and TTV

To detect their awareness of MNT and TTV, women were asked if they heard about MNT/TTV and the source of their information (if present). Knowledge was measured by seven statements assessing information about required doses and benefits of TTV, causes, symptoms, complications, and method of protection from MNT. Females responded to each statement by choosing among multiple given answers. The total score ranged from 0 to 7 points. The score was converted to a percentage and was divided into poor (less than 50%), fair (from 50 to 70%), and good knowledge (more than 70%).

#### HBM constructs

The scale measuring HBM constructs consists of 5 subscales measuring perceived susceptibility and severity of MNT, perceived benefits of and barriers to TTV, self-efficacy in receiving TTV, and cues to receiving the vaccine. Responses to scale items (except cues to action) were scored on a three-point Likert scale ranging from 0 (disagree) to 2 (agree). The score was reversed for some items. It was calculated for each subscale, converted to percentage, and categorized into high (> 66.67%), moderate (33.33 to 66.67%), and low (≤ 33.33%).

Cues to action were assessed by a single statement: “who is the person/source of information you trust his/her/its opinion regarding TTV?”

Total health belief: the scores of P. susceptibility, P. severity, P. benefits, and self-efficacy scales were summed and then the perceived barriers’ score was subtracted from the previous score to obtain the total health belief score.

### Statistical analysis

Data entry and statistical analysis were performed using SPSS version 21. Descriptive statistics such as percentages and frequencies were used to measure the demographic variables, immunity status**,** and the responses to knowledge, belief**,** and social support statements. Logistic regression analysis was performed to identify the significant predictors of tetanus toxoid vaccination practice. Statistical significance was set at *p* < 0.05.

## Results

Most of the studied women (79.1%) were of age 25 to less than 35 years with a mean age of 30 years (± 4 years). Most of them (79.3%) completed their secondary/university education and 53.6% were housewives. Nearly half of the husbands (46.9%) had secondary education, about one-third (32.4%) had university education or higher, and almost all of them (99.6%) were working. More than half of studied women (53.9%) were of moderate socio-economic level and 40% were of low socio-economic level.

All women received antenatal care in their last pregnancy; the vast majority of them (90.9%) received antenatal care in the private sector, while only a small percentage received antenatal care in maternal and child health centers (MCH) and governmental sector mainly in El-Shatby followed by Gamal Abd El-Naser then Aboquir hospitals (5.9% and 3.2% respectively) (Table 1).

**Table 1:** Demographic and last delivery characteristics of reproductive-age women in Alexandria, 2018

Sample characteristics (***n*** = 700)	Percent (%)	
Age of the mother		
< 25 years	9.3	
25–< 35 years	79.1	
35 + years (mean ± SD = 30 ± 4)	11.6	
Mother’s education		
Illiterate or just read and write	1.8	
Primary	2.9	
Preparatory	16.0	
Secondary	46.9	
University or higher	32.4	
Mother’s occupation		
Working	46.4	
Not working	53.6	
Husband’s education		
Illiterate or just read and write	3.0	
Primary	5.3	
Preparatory		13.4
Secondary		40.7
University or higher		37.6
Husband’s occupation		
Working		99.6
Not working		0.4
Socio-economic level		
Low	40	
Moderate	54	
High	6	
Antenatal care		
Yes	100	
No	0	
Place of antenatal care		
Maternal and child health centers	5.9	
Governmental sector	3.2	
Private sector		90.9

Nearly half of studied women (52.2%) delivered in the private sector, 45.4% in governmental hospital, and a very small percent (2.4%) delivered at home. Almost all deliveries were performed by obstetricians (94.6%) and the remaining percent were performed by nurses or midwives (data not presented).

**Fig. 1:**
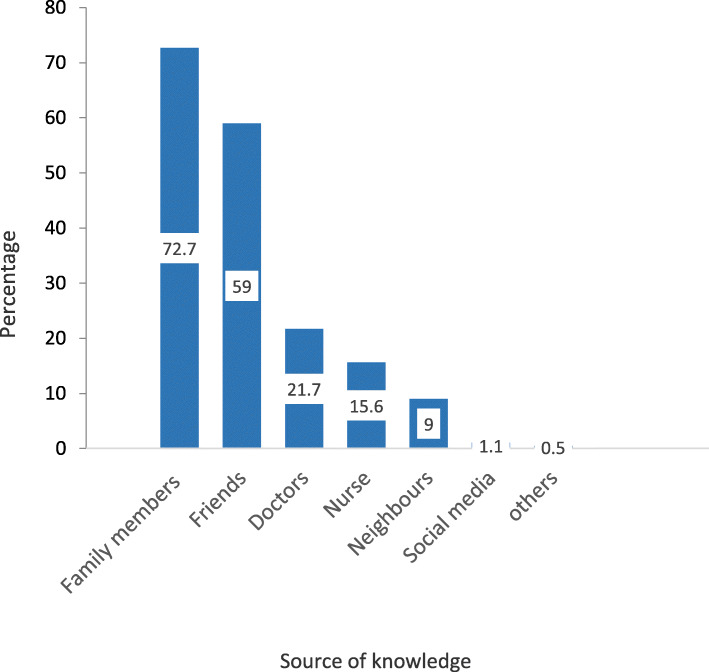
Source of information of reproductive-age women in Alexandria about TTV and MNT, 2018

The majority of studied women (79.0%) reported being aware about TTV/MNT. The main source of information was the family members (73.0%) and friends (59.0%) (Fig. 1).

Table 2 shows that the vast majority of women (89.7%) did not know or had incorrect information regarding the total number of TTV doses that should be taken during the reproductive age. More than one-third of them (37.9%) mentioned that the vaccine protected both the mother and her child. Furthermore, about half of them (47.0%) mentioned that TTV can protect from infections during delivery whatever the cause and only 8.9% recognized that this vaccine can protect from tetanus. Less than one-fourth of women (15.2%) reported delivery or abortion in non-equipped places as the main cause of MNT and around two-thirds of them (70.3%) mentioned that they did not know the exact causes of MNT. Moreover, most of them (87.6%) did not know MNT symptoms and around 95% did not know the complications of MNT. Delivery in equipped places and vaccination was mentioned by 16.2% and 11.1% of women, respectively, as methods of MNT prevention, while the about two-thirds did not know the methods of protection from tetanus.

**Table 2:** Knowledge of reproductive-age women in Alexandria about TTV and MNT, 2018

Knowledge (***n*** = 700)	Percent
Number of doses during the reproductive age*	
1 dose	0.1
2 doses	2.9
3 doses	3.5
4 doses	0.3
5 doses	3.6
Do not know	89.6
Person(s) protected by TTV	
Mother	21.5
Child	7.0
Mother and child	37.9
Do not know	33.6
TTV protects from	
Diphtheria	0.7
Puerperal sepsis	10.6
Maternal and neonatal tetanus	8.9
Toxoplasmosis	0.3
Infection during delivery	47.1
Pre-eclampsia	0.4
Premature labor	1.9
Prevent congenital anomalies	0.9
Jaundice after delivery	0.3
Do not know	28.9
Causes of maternal and neonatal tetanus	
Premature labor	1.1
Cutting umbilical cord by contaminated or rusted instruments	13.0
Delivery or abortion in un equipped places	15.2
Gestational diabetes	0.4
Do not know	70.3
Symptoms of maternal and neonatal tetanus	
Fever	3.6
Convulsions	0.6
Difficulty swallowing	0.2
Vomiting and diarrhea	1.0
All of above	5.4
Symptoms of MNT (cont’d)	
Other	1.6
Do not know	87.6
Complications of tetanus	
Maternal and neonatal death	5.6
Do not know	94.4
Methods of tetanus prevention	
Vaccination	11.1
Delivery in equipped places	16.2
Do not know	72.7

*2–5 doses are considered correct answers

**Fig. 2:**
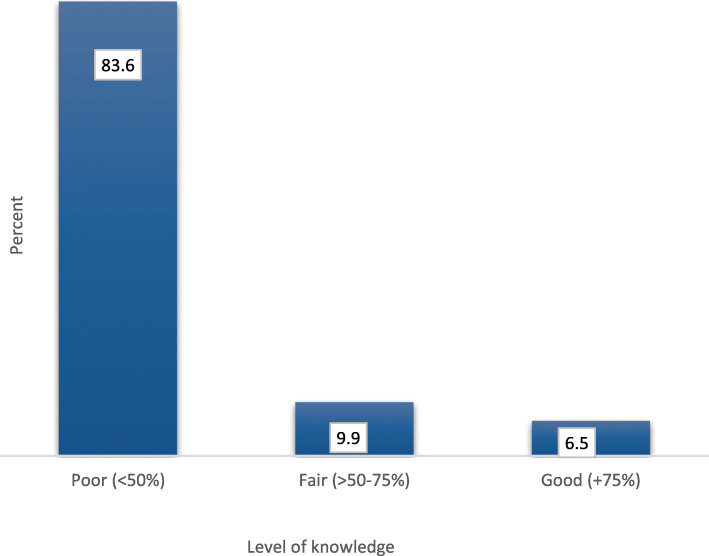
Level of knowledge of reproductive-age women in Alexandria regarding TTV and MNT, 2018

Figure 2 shows the overall percentage score of knowledge, where the majority of women (83.6%) had poor knowledge of MNT and TTV.

**Fig. 3:**
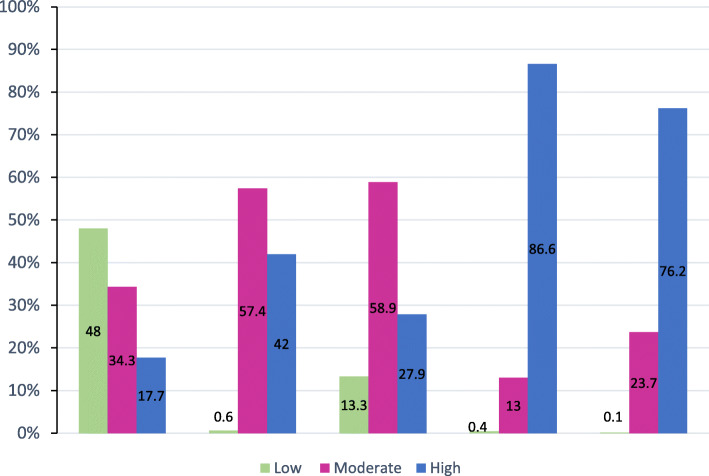
Health beliefs of reproductive-age women in Alexandria regarding TTV and MNT, 2018

Figure 3 shows that the highest percentage of women had low perception of susceptibility to MNT (48.0%), moderate perception of severity of MNT (57.4%) and barriers to TTV (58.9%), high perception of benefits of TTV (86.6%), and high self-efficacy in taking the vaccine (76.2%).

**Fig. 4:**
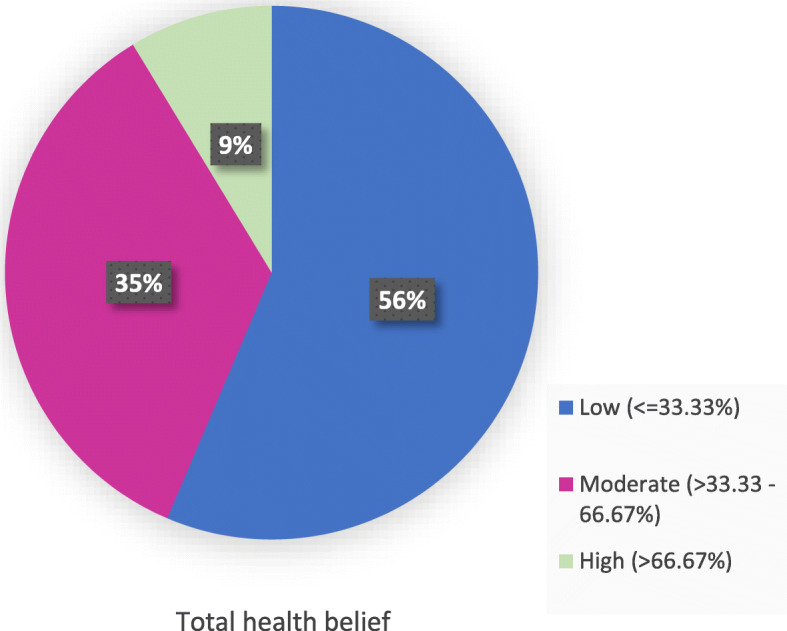
Total health belief level of reproductive-age women in Alexandria regarding TTV and MNT, 2018

Half of the women agreed that tetanus would not infect them if they had good immunity (50.1%) and were living in a clean place (51.3%) Only 17.9% agreed that they could be infected despite their delivery in equipped places. Although most women were not sure about the fatality of the disease (85.7%) or the possibility of it causing permanent disabilities to their child (88.3%), almost all of them agreed that even the thought of infection of their child worried them (96.7%). The most frequent benefit of taking TTV (88.3%) was feeling that they were performing their duties towards their children and the most agreed upon barriers were as follows: not knowing about TTV (73.1%), believing that vaccination may cause congenital anomalies or infertility (71.1% and 65.4%, respectively), and not knowing the place and appropriate timing for taking the vaccine (65.1%). More than two-thirds of women agreed that they could take the vaccine in time (73.6%) and by all doses (70.1%) whatever the barriers and under any circumstances tolerating the side effects (74.9%). The overall health belief percentage score was low for 56.4% of women (Table 3 and Fig. 4).

**Table 3:** Responses of reproductive-age women in Alexandria to the different belief items of the HBM, 2018

HBM constructs (*n* = 700)	Agree	Not sure	Disagree
	%	%	%
Perceived susceptibility			
I feel that me and my child are susceptible to tetanus.	23.7	52.9	23.4
Tetanus can affect me and my child despite my delivery in equipped places.	17.9	42.5	39.6
Tetanus can affect me and my child whatever the method of delivery (normal or CS).	20.3	69.6	10.1
If I am in good health and have strong immunity then I am not susceptible to the disease.	50.1	30.6	19.3
If I am living in a clean place with my child then I am not susceptible to disease.	51.3	31.7	17.0
Neonatal tetanus can affect my child under any circumstances even if I was careful.	13.0	44.0	43.0
Perceived severity			
Tetanus is severely dangerous for me and my child.	28.6	70.4	1.0
My child may die if affected by neonatal tetanus.	14.3	85.7	0.0
My child can have permanent disability if infected by neonatal tetanus.	10.0	88.3	1.7
I can face many difficulties if my child gets infected by neonatal tetanus.	38.7	61.0	0.3
Infection of my child with tetanus can affect my social relationship.	55.0	40.7	4.3
Even the thought of infection of my child by the disease makes me worried.	96.7	2.6	0.7
Perceived barriers			
I feel that the vaccination is not useful and effective.	64.0	20.0	16.0
I am busy and I have no time to go and take the vaccine.	32.9	23.3	43.8
I worry about the vaccine side effects.	14.6	13.0	72.4
I believe that vaccination during pregnancy may cause congenital anomalies.	71.1	23.7	5.2
I believe that vaccination may cause infertility.	65.4	27.5	7.1
I believe that vaccination may cause abortion if taken during pregnancy.	50.4	42.3	7.3
I do not know about tetanus vaccine.	73.1	10.0	16.9
I do not know the place and time of vaccination.	65.1	15.8	19.1
Tetanus vaccine is not always available.	30.7	43.4	25.9
I believe that the vaccine may cause the disease instead of preventing it.	18.6	74.3	7.1
The nurse who gives the vaccine is not always present.	25.9	53.0	21.1
I fear injections.	7.7	10.0	82.3
The behavior of the staff in health office is not good.	10.4	15.4	74.2
Perceived benefits			
The vaccine protects me and my child from tetanus.	73.3	26.4	0.3
Taking the vaccine is part of my duty towards my child.	88.3	11.3	0.4
Taking the vaccine makes me feel safe about myself and my child under any circumstances.	87.6	12.0	0.4
Self-efficacy			
I am confident I will take the vaccine in its time whatever the barriers.	73.6	26.3	0.1
I can commit to taking all doses of vaccine under any circumstances.	70.1	29.7	0.2
I am capable of getting all information I need about the vaccine.	72.4	27.6	0.0
I can tolerate the side effects of the vaccine.	74.9	24.2	0.9

**Fig. 5:**
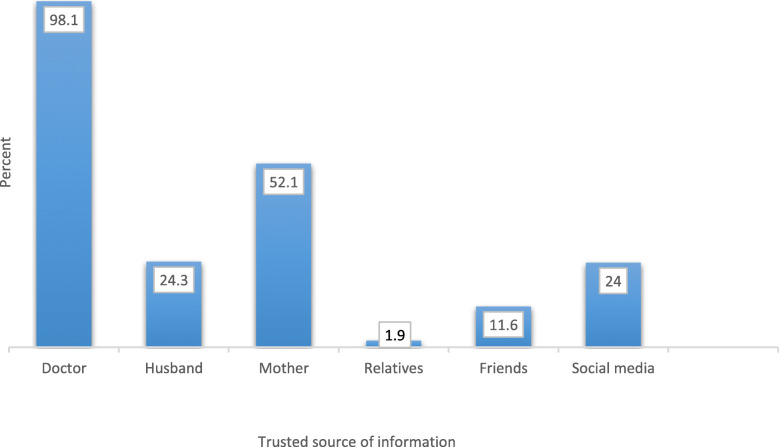
Cues to action of reproductive-age women in Alexandria regarding TTV, 2018

Regarding cues to action, almost all women (98.1%) agreed that doctor’s opinion was the most they trusted followed by mother’s opinion (50.1%) (Fig. 5).

More than half of the sampled women (59.0%) did not receive any dose of TTV and most of those who received the vaccine took only one or two doses (13.3% and 18.3% respectively). Less than one-third of the sampled women (27.7%) were immune by (TT2+) (Table 4).

**Table 4:** Vaccination status of reproductive-age women in Alexandria, 2018

Vaccination status (*n* = 700)	%
Receiving tetanus toxoid vaccine in last pregnancy or any previous pregnancies	41.0
Number of doses received*	
1 dose	13.3
2 doses	18.3
3 doses	7
4 doses	2
5 doses	0.43

*Percent is calculated from total (*n* = 700)

Tables 5 and 6 show the logistic regression analysis of immunity status and some independent variables. Two models were used; the first model included subtotal scores and total knowledge score and the second included total scores as independent variables.

**Table 5:** Multivariate logistic regression analysis of immunization status of reproductive-age women in Alexandria in 2018 with subtotal scores

Predictor	Multivariate				
	B	S.E.	OR	95% CI	*p* value
Place of antenatal care					
Maternal and child health centers^(R)^					0.008
Governmental sector	− 1.766	0.660	0.171	0.047–0.623	0.007
Private sector	− 1.385	0.474	0.250	0.099–0.634	0.003
Total knowledge grade					
Good^(R)^					0.032
Poor	− 0.833	0.363	0.435	0.213–0.886	0.022
Fair	− 0.333	0.423	0.717	0.313–1.644	0.432
Score of perception of barriers					
High^(R)^					0.011
Low	0.990	0.338	2.692	1.387–5.224	0.003
Moderate	0.617	0.262	1.854	1.109–3.098	0.018
Constant	1.451	0.895	4.269	–	0.105

*R* reference group, *OR* Odds Ratio

**Table 6:** Multivariate logistic regression analysis of immunization status of reproductive-age women in Alexandria in 2018 with total scores

Predictor	Multivariate				
	B	S.E.	OR	95% CI	*p* value
Place of antenatal care					
Maternal and child health centers^(R)^					0.003
Governmental sector	− 1.743	0.630	0.175	0.051–0.601	0.006
Private sector	− 1.490	0.449	0.225	0.093–0.544	0.001
Total health belief score					
High^(R)^					0.066
Low	− 0.690	0.350	0.502	0.253–0.996	0.049
Moderate	− 0.303	0.338	0.738	0.380–1.433	0.370
Socio-economic level					
Low^(R)^					0.001
Moderate	0.648	0.406	1.911	0.862–4.235	0.111
High	− 0.113	0.430	0.893	0.384–2.076	0.793
Constant	2.3351	0.807	10.497	–	0.004

*R* reference group, *OR* Odds Ratio

After adjusting for mother’s and father’s education and occupation, the place of last delivery, and perceived severity score, it was found that the significant predictors of immunity were as follows: the place of antenatal care, level of knowledge, and perceived barriers (*p* = 0.008, 0.032, and 0.011, respectively). While antenatal care in MCH centers was a significant predictor of positive immunity status, ANC in governmental sector resulted in 17.1% decrease in the probability of being immune (OR = 0.171, 95% CI 0.047–0.623, *p* = 0.007) and that ANC in the private sector decreased probability of being immune by 25% (OR = 0.250, 95% CI 0.099–0.634, *p* = 0.003). Having poor knowledge resulted in 43.5% decrease in the probability of being immune (OR = 0.435, 95% CI 0.213–0.886, *p* = 0.022). Moderate and low perception of barriers resulted in 18.5% and 26.9% increase of the probability of being immune respectively (OR = 2.692, 95% CI 1.387–5.224, *p* = 0.003 and OR = 1.854, 95% CI 1.109–3.098, *p* = 0.018 respectively) (Table 4).

In the second model (Table 5), the place of delivery, place of antenatal care, total knowledge, total health belief scores, and socio-economic score were entered as predictor variables. Place of antenatal care (*p* = 0.003) and socio-economic level (*p* = 0.001) emerged as significant predictors of immunity status, while level of knowledge (*p* = 0.07) and total health belief (*p* = 0.06) were not. However, total health belief approached significance where low health belief resulted in 50.2% decrease in the probability of being immune (OR = 0.502, 95% CI 0.253–0.996, *p* = 0.049).

## Discussion

This study focused on defining the knowledge and health beliefs of Egyptian women in Alexandria regarding tetanus toxoid vaccination. Although TTV intake among these women was relatively high (41%), only 27.7% were immune (achieved TT2+ injections as recommended by WHO). This figure is much lower than those identified in other studies in Egypt, Cairo [15] and Dakahlia [14] governorates (42.6% and 63.2% respectively), and outside Egypt, in Pakistan (40.4 to 65%) [20] and in Ethiopia (39.2% of all child-bearing age women living in Dukem town) [21]. This low uptake of TTV among women in Alexandria may be attributed to different factors such as the following: poor knowledge of the majority of the studied women about MNT and/or TTV, their low perception of being susceptible to MNT, perceiving several barriers to TTV, including the misconception that the vaccine could result in congenital anomalies for babies, and the influence of health care providers. This finding sheds strong light on the passive or even negative role of obstetricians in Alexandria regarding TTV.

Findings of this study revealed that having good knowledge was a significant predictor of being immune. Similarly, findings from a study in Iraq (2014) [22] and another in Pakistan (2010) [23] showed that most of the women who were immune had good knowledge. It is worth noting that although most women in our study were aware about MNT/TTV, they had poor knowledge about some vaccine and/or disease-related information such as the number of doses of TT vaccine needed to be taken, the disease prevented by TTV, and the clinical picture of MNT. Consistent results were reported by Dhia et al. [24]. Such findings reflect the shortage in information delivered by medical personnel to these women whose source of information was mainly family members and friends, while the role of obstetricians, nurses, and media was not pertinent.

A significant relationship was found between TTV intake and socio-economic level in the current study and in a previous Egyptian study conducted in 2016 [15]. Our findings showed that low socio-economic level was a significant predictor of being immune (TTV2+), which gives a hint that different messages are given to pregnant women according to their socio-economic level and intended place of delivery, where women who were expected to deliver in non-equipped hospitals were advised to take the vaccine, while those who were expected to deliver in relatively well-equipped hospitals are advised otherwise. It seems that obstetricians themselves hold a false concept that as long as delivery occurs in equipped and clean places, then, there is no need for the vaccine. In fact, this argument would only be valid in a situation where all deliveries are performed under ideal conditions and when effective tetanus immunization programs and good post-exposure prophylaxis outside pregnancy exist, which is hardly the case in most developing countries [25].

In the present study, receiving ANC in MCH centers appeared to be a significant predictor of positive immunity status. ANC service offered in MCH centers is usually used by women belonging to low or middle social classes. It seems that obstetricians in MCH centers work according to a clinical protocol, which includes motivating pregnant women to take the vaccine. Different findings were obtained by Roosihermiatie et al. [26], where women who received antenatal care, no matter the place, were 30 times more likely to be immune than those who did not. This again draws attention to the negative messages given by obstetricians in the private sector regarding the importance of receiving the vaccine. When obstetricians’ opinions were shown to be the most important cue to taking the vaccine, some women revealed that their obstetricians discouraged them from taking it.

The HBM was used in this study for a clearer understanding of the possible link between health beliefs and immunization behavior. Literature has underscored the role of perceived barriers as an important predictor of health behavior [12, 13, 27–30]. Findings of the current study lend support to previous research where of all constructs of the HBM, perceived barriers were the only significant predictor of immunity status in which moderate and low perception of barriers resulted in 18.5% and 26.9% increase of probability of being immune, respectively. Several barriers were documented in the current study such as the following: not knowing about TTV, unavailability of vaccine or personnel giving this service, and believing that vaccination may cause congenital anomalies or infertility. These barriers need to be appropriately addressed via different communication channels including face to face and media channels.

In accordance with our findings, a study conducted in Malawi (2013) [31] demonstrated that women having low perception of barriers were most likely to be immune (75%) followed by those having moderate perception (15%), while women having high perception of barriers were the least likely to be immune (10%).

These findings should direct our efforts to the priority areas needed to be addressed in educating women, which are knowledge about the disease and vaccine and perceived barriers to taking the vaccine. It is worth noting that the emphasis on some HBM constructs does not mean that we should ignore other constructs to achieve synergetic effect given that women’s total health belief approached significance (*p* = 0.06) in logistic regression analysis, where low health belief resulted in 50.2% decrease in the probability of being immune.

The role of total health belief in being immune against MNT was further illustrated by a study in Nigeria (2017) showing that the majority of women with low health belief were not immune [32].

However, these results were contradicted by the findings of Vandelaer et al. [33], where 72.6% of immune women had low health belief level. Perhaps these women were obliged to take the vaccine as a part of the policy of ANC applied in their country regardless of their beliefs about the disease or vaccine.

### Limitations of the study

This study has some limitations. First, because this study is cross-sectional, a temporal relation between exposure and outcome cannot be established given the fact that data were collected for current health beliefs and prior immunization behavior. Longitudinal data are needed to verify the results observed in this study. Second, data collection was based on an interview questionnaire and only a small percent of participants who reported receiving TTV had the vaccination card; thus, data might have been subjected to information bias (recall bias). Moreover, other reasons for some obstetricians’ discouragement of women to take the vaccine such as inadequate training, inadequate reimbursement, and increased workload were not addressed in this work. These areas should be addressed in future research.

## Conclusion

This study indicated that the use of TTV among females in child-bearing age in Alexandria was low. The theoretical framework of the widely accepted HBM has provided an important insight into women’s beliefs of MNT/TTV. Barriers to receiving tetanus toxoid vaccination were shown to be an important predictor of immunization behavior along with place of antenatal care and mothers’ knowledge about TTV and MNT. Our findings shed important light on the lack of appropriate MNT/TTV information and the discouragement women receive from their private obstetricians in their ANC visits. The results of this study should be seriously considered in the development of relevant health care policies and clinical protocols to enhance TTV immunization among females in child-bearing age.

## Data Availability

Data and questionnaire are available from the corresponding author upon request.
